# Tabanids as possible pathogen vectors in Senegal (West Africa)

**DOI:** 10.1186/s13071-020-04375-w

**Published:** 2020-10-01

**Authors:** Mohamed Lamine Keita, Hacène Medkour, Masse Sambou, Handi Dahmana, Oleg Mediannikov

**Affiliations:** 1 IHU Méditerranée Infection – Microbes, Evolution, Phylogeny and Infection (MEФI), Marseille, France; 2 UMR Aix-Marseille University, IRD, APHM -19-21, Bd Jean Moulin, 13385 Marseille Cedex 05, France; 3 IHU Méditerranée Infection - Vecteurs - Infections Tropicales et Méditerranéennes (VITROME), Marseille, France; 4 Vectors-Tropical and Mediterranean Infections (VITROME), Campus International, UCAD-IRD, Dakar, Sénégal

**Keywords:** Tabanids, *Leishmania donovani*, *Trypanosoma* spp., PCR, MALDI-TOF MS, Senegal

## Abstract

**Background:**

Species of the Tabanidae are potent vectors of human and animal diseases, but they have not been thoroughly investigated to date. In Senegal (West Africa), little information is available on these dipterans. Our objective in this study was to investigate Senegalese tabanids and their diversity by using molecular and proteomics approaches, as well as their associated pathogens.

**Methods:**

A total of 171 female tabanids were collected, including 143 from Casamance and 28 from Niokolo-Koba. The samples were identified morphologically by PCR sequencing and by MALDI-TOF MS, and PCR analysis was employed for pathogen detection and blood-meal characterization.

**Results:**

The morphological identification revealed four species concordantly with the molecular identification: *Atylotus fuscipes* (79.5%), *Tabanus guineensis* (16.4%), *Chrysops distinctipennis* (3.5%) and *Tabanus taeniola* (0.6%) (not identified by PCR). The molecular investigation of pathogens revealed the presence of *Trypanosoma theileri* (6.6%), *Leishmania donovani* (6.6%), *Setaria digitata* (1.5%), *Rickettsia* spp. (5.1%) and *Anaplasmataceae* bacteria (0.7%) in *A. fuscipes*. *Tabanus guineensis* was positive for *L. donovani* (35.7%), *S. digitata* (3.6%) and *Anaplasmataceae* (17.8%). *Leishmania donovani* has been detected in 50% of *C. distinctipennis* specimens and the only *T. taeniola* specimen. No Piroplasmida, *Mansonella* spp. or *Coxeilla burnetii* DNA was detected. In addition to humans (96.43%), *Chlorocebus sabeus*, a non-human primate, has been identified as a host of (3.57%) analysed tabanids. MALDI-TOF MS enabled us to correctly identify all tabanid species that had good quality spectra and to create a database for future identification.

**Conclusions:**

Tabanids in Senegal could be vectors of several pathogens threatening animal and public health. To fully characterize these dipterans, it is therefore necessary that researchers in entomology and infectiology employ molecular characterization and mass spectrometric techniques such as MALDI-TOF MS to analyse these dipterans in Senegal and West Africa.
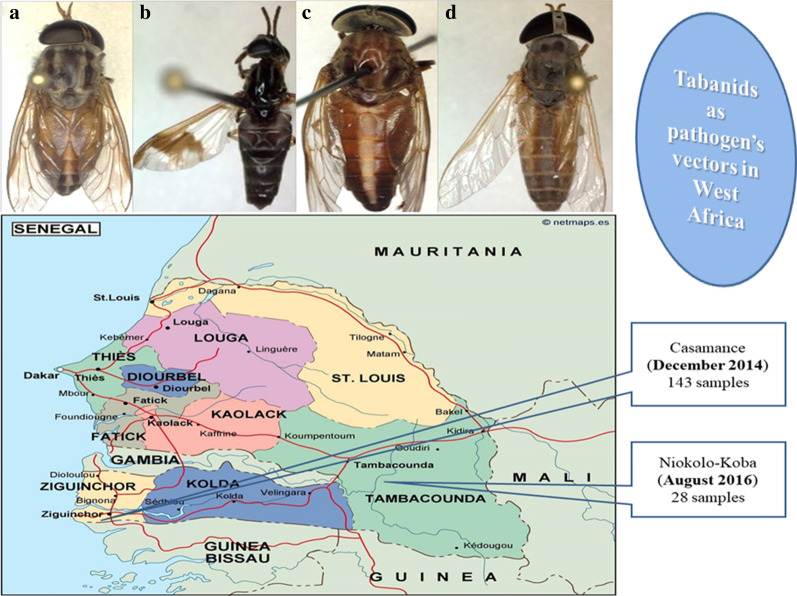

## Background

The Tabanidae is an important dipteran family belonging to the infraorder of Tabanomorpha and to the superfamily Tabanoidae [1, 2]. There are 4400 species grouped in 114 genera, distributed in four subfamilies: Chrysopsinae; Pangoninae; Tabaninae; and Scepsidinae [2–4]. In Africa, only the first three subfamilies are of economic and medical interest due to their impact on livestock and their role as potential vectors of diseases [1, 2, 5]. Tabanidae have a global distribution, including 410 species in South Africa, 335 in North America, 244 in India, 120 in Malaysia and nearly a hundred in Thailand [1, 3–6]. Male insects feed on nectar and do not present medical interest. In contrast, female tabanids of most species are haematophagous, being able to obtain large stores of blood from their hosts by telmophagy [1, 2]. These arthropods are considered to be primarily mechanical vectors of various human and animal pathogens, including bacteria, viruses, helminths, and protozoans [2, 6, 7]. Foil et al. [8] identified 25 different diseases transmitted by these dipterans. Krinsky [9] listed 189 species of the Tabanidae with approximately 30 infectious agents. Despite this role, the Tabanidae remain among the least-studied dipteran species [2]. To date, our research on PubMed using “Tabanidae” as a keyword yielded only 382 references, including 200 publications in 1976 by Krinsky [8] and 46 for African Tabanidae and 182 publications over 43 years.

To prevent damage from the Tabanidae and to elucidate their vectorial roles in the transmission of infectious diseases, correctly identifying their species and associated pathogens has always been necessary [3, 5]. This identification is based on both morphological and molecular tools [7, 10]. Nonetheless, the infectious status and hosts associated with these species can only be determined by molecular biology [11, 12]. Morphological identification has the advantage of being fast and economical but requires expertise from entomologists. This method may present such difficulties as alteration of specimens, variations in developmental stages and morphological similarities [1, 3].

The molecular identification of species proposed by Hebert et al. [3] is based on the amplification of 658 bp of the mitochondrial gene coding for cytochrome *c* oxidase subunit 1 (*cox*1) . This method, called “barcoding”, enables the discrimination of a wide range of species and offers the advantage of integrating taxonomic and genomic data, regardless of genital or developmental status [5, 13]. In addition, one of the fundamental tasks of barcoding is to associate genomic sequences to the species names [14]. Furthermore, this method makes it possible to reveal the genetic diversity and to solve the ambiguities at the species level with precision, constancy and celerity [5]. Also, the molecular characterization of pathogens was largely based on the amplification of conserved ribosomal gene subunits [15]. However, there are some limitations for molecular identification, such as the scarcity of sequences on GenBank DNA database, particularly with regard to the Tabanidae.

The MALDI-TOF/MS technique is another more recent arthropod identification approach but is developing rapidly in terms of cost, time, sensitivity, specificity and reproducibility [16]. Unlike molecular analysis, this technique relies on the production and analysis of protein spectra specific to each species, regardless of the stage of development [17]. Abdomen, thorax or limbs may be used for identification [17]. The difficulty with MALDI-TOF/MS is the lack of databases and reference spectra [16, 18]. Additionally, the use of certain parts of arthropods, such as the abdomen, containing exogenous proteins of various hosts influences the quality of spectra [17]. One of the objectives of the present study was to initiate a MALDI-TOF/MS database for the Tabanidae that has not been available to date.

In the Republic of Senegal, in West Africa, livestock farming is one of the major activities, particularly in rural areas. Vector-borne diseases, such as trypanosomiasis and filariasis, are endemic [19–21]. To date, 43 species of the Tabanidae belonging to the genera *Atylotus*, *Chrysops*, *Ancala*, *Hybomitra*, *Haematopota*, *Philoliche* and *Tabanus* have been reported [22]. A series of entomological works conducted by a generation of researchers, including Oldroyd [23, 24], Ovazza and Valade [25] and Raymond et al. [22], made it possible to perform a morphological description and study the taxonomy of this notably diverse fauna. The recurrent presence of HIV, tuberculosis, malaria or dengue fever since the 1980s has diverted researchers from investigating tabanids in Senegal. Except for some studies, such as Touré’s in 1968 on cattle trypanosomiasis in Casamance [26], knowledge on pathogens carried by tabanids in Senegal is non-existent and marked by a scarcity of bibliographic resources.

Moreover, despite the existence of vector-borne diseases, such as filariasis and trypanosomiasis, to the best of our knowledge, no reports have described the molecular and proteomics identification of tabanid flies in Senegal. The aim of this study was to identify samples of the Tabanidae from Senegal and characterize their associated pathogens and hosts.

## Methods

### Study sites

Sample collection was carried out at two sites in southern Senegal: Lower Casamance in December 2014 and Niokolo-Koba National Park in August 2016.

Lower Casamance is located in southern Senegal and is isolated from the rest of the country by Gambia. Ziguinchor, the capital of the region, borders the Casamance River. The Lower Casamance covers an area of 7339 km^2^ and is composed of three departments: Ziguinchor (regional capital), Bignona and Oussoye. The catches took place in several localities of the department of Oussoye. The climate is tropical of type subguineen, characterized by a rainfall of approximately 1500 mm/year, as well as a high hygrometry related to both the marine influence and a low thermal amplitude.

Niokolo-Koba National Park (PNNK) is a park located 650 km east of Dakar in the region of Tambacounda (southeastern Senegal) near the Guinean border. The Gambia River runs through the park, as well as its two tributaries: the Koulountou River and the Niokolo-Koba River, which gave the park its name. The relief is quite flat with an altitude of 16–311 m. Mount Assirik is the highest point. Large plains, sometimes marshy in the rainy season, separate small hills of barely 200 m. The terrain becomes more rugged in the extreme southeast on the foothills of the Fouta-Djalon mountain range where the Niger, Senegal and Gambia rivers originate. These foothills form a natural border between Senegal and Guinea. The Simenti pond, the site of our collection, is the only permanent pond in the park and attracts savannah animals that drink very salty water and roll in the mud.

### Methods and period of collection

Lower Casamance tabanids were caught using tsetse monocone traps. Ten traps were placed at 6:00-12:00 h the morning between pastures and houses, close to water points. Those of Niokolo-Koba were collected manually around camp lights during the evening. Samples were pooled by location and day of collection and then stored in 70% ethanol at room temperature until morphological identification was performed.

### Morphological identification

**Fig. 1:**
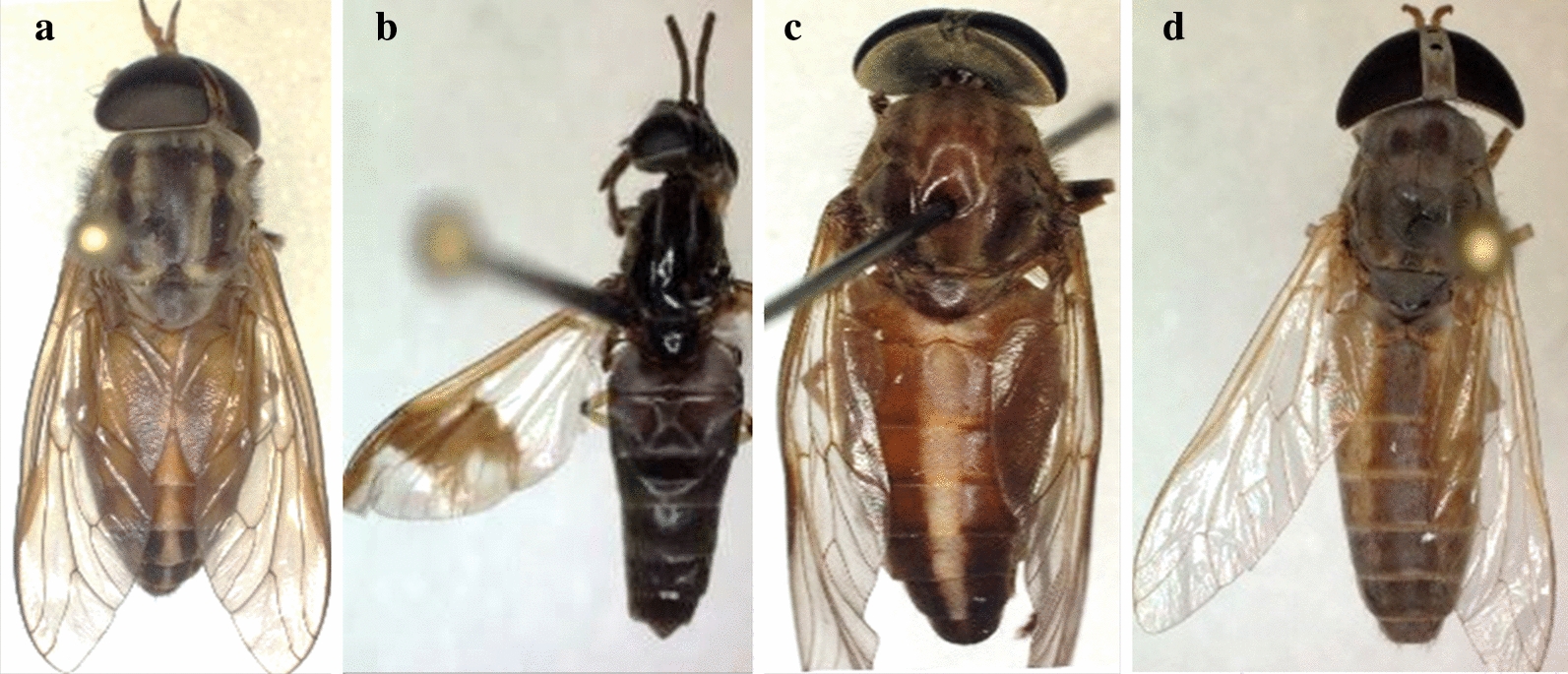
Morphotypes of the collected tabanid species. **a** *Tabanus taeniola.* **b** *Chrysops distinctipennis*. **c** *Tabanus guineensis*. **d** *Atylotus fuscipes*

All samples were subjected to double morphological identification; first, at the genus level in the collection sites, then at the species level in the University Hospital Institute-Méditerranée Infection insectarium, Marseille, France, using the Zeiss Axio Zoom V16 microscope (ZEISS, Oberkochen, Germany) and according to the different characteristics described by Oldroyd [23, 24]. Pictures were taken on three specimens of each species (Fig. 1).

### Dissection and sample preparation

The samples were rinsed with RNase-free water to eliminate ethanol and dissected longitudinally into two equal parts. One half was used for the molecular characterization of pathogens, species and hosts and the other half was stored in 70% ethanol for further analysis. Legs were used for analysis by MALDI-TOF MS; we preferred the legs to avoid interference during identification due to non-specific exogenous proteins from tabanids. These proteins are found in some parts of arthropods and generally affect the quality of MALDI-TOF MS spectra [27].

### DNA extraction

DNA extraction was performed using the QIAamp DNA Mini Kit® (Qiagen, Courtaboeuf, France) on BIOROBOT EZ1 (Qiagen) according to the manufacturer’s protocol.

### Molecular identification of Senegalese tabanids and their hosts

To confirm the morphological identification of the tabanids, a standard PCR targeting the *cox*1 gene using LCO/HCO primers developed by Folmer et al. (Table 1) was performed. The reaction mixture was 25 μl per reaction, including 5 μl of tabanid DNA template and 20 μl of mix (12.5 μl of Amplitaq Gold Master Mix; Eurogentec, Liège, Belgium; 6 μl of RNase-DNase-free water and 0.75 μl of each primer). No positive controls were used. After visualization by electrophoresis on a 2% agarose gel, the PCR product was purified using NucleoFast 96 PCR plates (Macherey Nagel EURL, Hoerdt, France) according to the manufacturer’s instructions. The amplicons were sequenced using the Big Dye Terminator Cycle Sequencing Kit (Perkin Elmer Applied Biosystems, Foster City, CA, USA) with an ABI automated sequencer (Applied Biosystems). The obtained electropherograms were assembled and edited using ChromasPro software (ChromasPro 1.7, Technelysium Pty Ltd., Tewantin, Australia) and checked against the GenBank database by NCBI BLASTn (https://blast.ncbi.nlm.nih.gov/Blast.cgi).

**Table 1:** PCR systems used in this study and their sources

Targeted organism	Target gene	Name	Primers (5′–3′) and probe	Tm* (°C)	Reference
Tabanidae	*cox*1	LCO1490	GGTCAACAAATCATAAAGATATTGG	50	[49]
		HCO2198	TAAACTTCAGGGTGACCAAAAAATCA		
Hosts	*16S*	16SA-2290	CGCCTGTTTACCAAAAACAT	50	[50]
		16SB-2860	CCGGTCTGAACTCAGATC ACGT		
*Leishmania donovani* complex	kDNA	RV1	CTTTTCTGGTCCTCCGGGTAGG	60	[51]
		RV2	CCACCCGGCCCTATTTTACACCAA		
		Probe. Leish*	FAM-TTTTCGCAGAACGCCCCTACCCGC-TAMRA		
*Leishmania* spp.	ITS1	rDNA-10 F	CAATACAGGTGATCGGACAGG	55	[52]
		rDNA-14 R	CACGGGGATGACACAATAGAG		
Piroplasmida	*5.8S*	5.8S-F5	AYYKTYAGCGRTGGATGTC	60	[53]
		5.8S-R	TCGCAGRAGTCTKCAAGTC		
		5.8S-S*	FAM-TTYGCTGCGTCCTTCATCGTTGT-MGB		
*Trypanosoma* spp.	*5.8S*	S. 5.8 S Tryp sp.	FAM-GTTGAAGAACGCAGCAAAGIGCGAT-TAMRA	60	[37]
		F. 5.8 S Tryp sp.	CAACGTGTCGCGATGGATGA		
		R. 5.8 S Tryp sp.	ATTCTGCAATTGATACCACTTATC		
	*28S*	F2 28S	ACCAAGGAGTCAAACAGACG	58	
		R1 28S	GACGCCACATATCCCTAAG		
	ITS1	ITS1-CF	CCGGAAGTTCACCGATATTG	58	[54]
		ITS1-BR	TTGCTGCGTTCTTCAACGAA		
Kinetoplastida spp.	*28S*	F2 28S	ACCAAGGAGTCAAACAGACG	58	[55]
		R1 28S	GACGCCACATATCCCTAAG		
	*18S*	F 720	GTTAAAGGGTTCGTAGTTGAA	50	This study
		R1495	GACTACAATGGTCTCTAATCA		
*Coxiella burnetii*	IS1111	F	CAAGAAACGTATCGCTGTGGC	60	[56]
		R	CACAGAGCCACCGTATGAATC		
		S	FAM- CCGAGTTCGAAACAATGAGGGCTG-TAMRA		
*Anaplasmataceae*	*23S*	TtAna-F	TGACAGCGTACCTTTTGCAT	60	[57]
		TtAna-R	GTAACAGGTTCGGTCCTCCA		
		TtAna-S*	FAM-CTTGGTTTCGGGTCTAATCC-TAMRA		
*Borrelia* spp.	*16S*	Bor_16S_3F	AGCCTTTAAAGCTTCGCTTGTAG	60	[58]
		Bor_16S_3R	GCCTCCCGTAGGAGTCTGG		
		Bor_16S_3P	FAM- CCGGCCTGAGAGGGTGAACGG-TAMRA		
*Ricketssia* spp.	Citrate synthase (gltA)	RKNDO3_F	GTGAATGAAAGATTACACTATTTAT	60	[46]
		RKNDO3_R	GTATCTTAGCAATCATTCTAATAGC		
		RKNDO3_S	FAM-CTATTATGCTTGCGGCTGTCGGTTC-TAMRA		
	*gltA* (First PCR)	CS2D	ATGACCAATGAAAATAATAAT	54	[59]
		CSEndR	CTTATACTCTCTATGTACA		
	*gltA* (nested PCR)	409D	CCTATGGCTATTATGCTTGC	54	
		1258R	ATTGCAAAAAGTACAGTGAACA		
*Mansonella* spp.	ITS	Manso_ITS_F	CCTGCGGAAGGATCATTAAC	60	[20]
		Manso_ITS_R	ATCGACGGTTTAGGCGATAA		
		Manso_ITS_P	FAM- CGGTGATATTCGTTGGTGTCT-TAMRA		
Filariae	*18S*	Fwd.18S.631	TCGTCATTGCTGCGGTTAAA	54	[60]
		Rwd.18S.1825r	GGTTCAAGCCACTGCGATTAA		

An identical process using primers targeting the vertebrate-specific *16S* rRNA gene was performed to identify hosts (Table 1). The *Agama agama* DNA extract from the IHU collection represented the positive control. The molecular identification involved 6 specimens of *Atylotus fuscipes*, 6 of *Tabanus guineensis*, 6 of *Chrysops distingutipennis* and the only specimen of *T. taeniola*.

### Pathogen study

Real-time quantitative PCR (qPCR) was used in the initial screening for pathogens. Reactions were performed in a CFX96 Real-Time system (Bio-Rad Laboratories, Foster City, CA, USA) according to the manufacturer’s instructions. The reaction mixture consisted of 5 μl of DNA and 15 μl of mix, including 10 μl Eurogentec Master Mix (Eurogentec, Liège, Belgium), 0.5 μl of each primer and uracile-DNA glycosylase (UDG), and 0.5 μl of the FAM-labelled probe. qPCR amplification was performed using the following thermal profile: incubation at 50 °C for 2 min for UDG action (eliminating PCR amplicon contaminants), followed by an activation step at 95 °C for 3 min, followed by 40 cycles of denaturation at 95 °C for 15 s and annealing-extension at 60 °C for 30 s.

The 171 specimens of the Tabanidae were screened using qPCR systems targeting the following genes: *5.8S* rRNA for *Piroplasmida*; IS1111 for *Coxiella burnetii*; *28S* rRNA for Filaroida; *5.8S* rRNA for *Trypanosoma* spp.; kinetoplast DNA (kDNA) for *Leishmania donovani* complex (*L. donovani* and *L. infantum*), ITS1 for *Mansonella* spp.; citrate synthase (*gltA*) specific for *Rickettsia* spp.; and *23S* rRNA for *Anaplasmataceae* (Table 1). Then, a standard PCR amplifying larger amplicons followed by sequencing was carried out for the qPCR-positive samples (Table 1). PCR assays and sequencing were performed as described above. In addition, semi-nested PCR was applied to optimize the detection capacity for Filaroida and *Rickettsiae*. The primers and probes used in this study are listed in Table 1.

### Phylogenetic analysis

The obtained sequences for the Tabanidae and their associated pathogens were assembled and edited as mentioned above. Then, the sequences were compared to sequences available on GenBank (https://blast.ncbi.nlm.nih.gov/Blast.cgi). Molecular phylogenetic and evolutionary analyses were conducted in MEGA7 (https://www.megasoftware.net/) using the maximum likelihood (ML) or neighbour-joining (NJ) methods with 100 replicates.

### MALDI-TOF MS analysis

Tabanids were individually rinsed with distilled water, and the 6 legs were removed and homogenized in an Eppendorf tube containing a pinch of glass powder (Sigma-Aldrich, Lyon, France) and 40 μl of a 70% (v/v) formic acid mixture and 50% (v/v) acetonitrile (Fluka, Buchs, Switzerland) using the TissueLyser device (Qiagen, Hilden, Germany) and following configuration parameters as previously described [18].

The homogenized tabanid legs were centrifuged at 2000× *g* for 30 s, and 1 μl of the supernatant from each sample was carefully dropped onto the MALDI-TOF target plate. Each spot was then recovered with 1 μl of matrix solution composed of saturated α-cyano-4-hydroxycynnamic acid (Sigma-Aldrich), 50% acetonitrile (v/v), 2.5% trifluoroacetic acid (v/v) (Sigma-Aldrich, Dorset, UK) and HPLC-grade water. The target plate, after several minutes of drying at room temperature, was introduced into the Microflex LT MALDI-TOF Mass Spectrometer device (Bruker Daltonics, Bremen, Germany) for analysis. The loading of the MS target plate, the matrix quality and the performance of the MALDI-TOF were performed as previously described [18]. The obtained MALDI-TOF MS spectra were analysed using Flex 3.3 analysis software and ClinPro-tools 2.2. Only good-quality spectra were selected according to their intensity and reproducibility for subsequent analyses. The spectra of two specimens of *T. guineensis*, *C. distinctipennis* and *A. fuscipes* were introduced into the database. These species have been previously identified by morphology and confirmed by molecular biology (except for *T. taeniola*, which could not be identified molecularly). The spectra of the remaining specimens were used for the blind test to finally assess the ability of MALDI-TOF MS to identify them.

## Results

### Collection and morphological characterization of tabanids

A total of 171 samples of the Tabanidae were captured. Due to the quality of the bait, all the specimens were females, including 143 in Casamance and 28 in Niokolo-Koba. The morphological identification revealed three species in Casamance, including *A. fuscipes* Ricardo, 1908 for 136 samples (95%), *C. distinctipennis* Austen, 1906 for 6 samples (4%) and *T. taeniola* Palisto de Beavios 1807 for 1 sample (0.69%). Niokolo-Koba samples consisted only of *T. guineensis* Wiedemann, 1824 (*Tabanus subangustus* Ricardo, 1908), 28 samples (100%) [28]. In the present study, the abundant species was *A. fuscipes* (79.5%).

### Molecular characterization and phylogeny of the Tabanidae

**Fig. 2:**
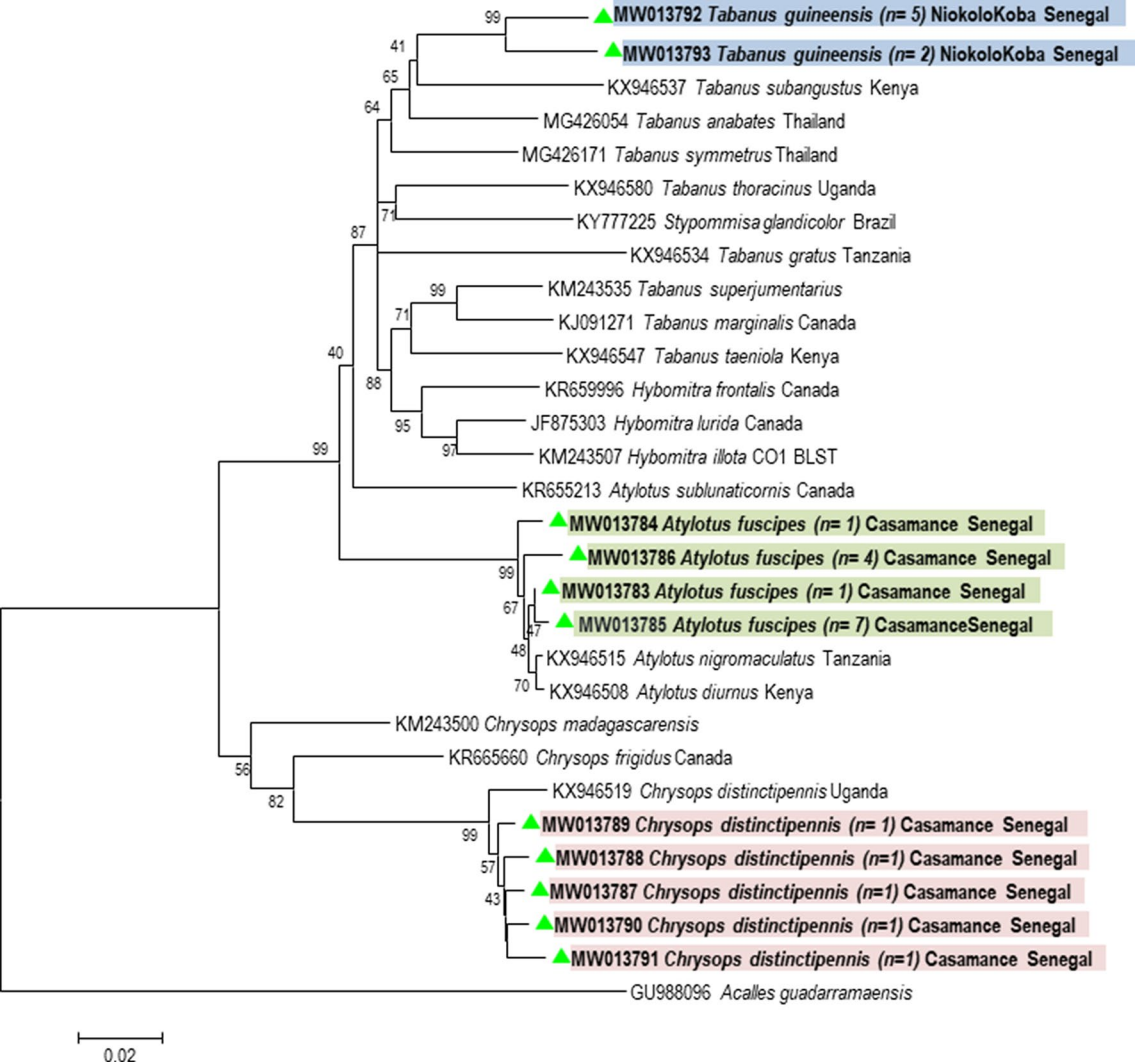
Phylogenetic tree highlighting the position of the tabanid species identified in the present study compared to other sequences available on GenBank. The evolutionary history, based on the *cox*1 gene, was inferred using the neighbour-joining method. The evolutionary distances were computed using the Tamura-Nei method and are in units of the number of base substitutions per site. The differences in the composition bias among sequences were considered in evolutionary comparisons. The analysis involved 30 nucleotide sequences. All positions containing gaps and missing data were eliminated. There were a total of 655 positions in the final dataset

BLAST analysis of the c*ox*1 sequences revealed that *T. guineensis* specimens from Niokola-Koba exhibited approximately 93% identity with *Tabanus subangustus* described in East Africa (Kenya) (GenBank: KX946537) [28]. *Atylotus fuscipes* had 99% identity with *A. nigromaculatus* (GenBank: KX946515) and *A. diurnus* (GenBank: KX946508) from East Africa. The identity of *C. distingutipennis* was up to 98% with that described in Uganda (GenBank: KX946519). We were unable to confirm the molecular identity of *T. taeniola* due to the poor quality of the sequences. The phylogenetic analysis using the neighbour-joining method revealed the existence of three clades: Clade 1 (*Tabanus* spp.); Clade 2 (*Atylotus* spp.); and Clade 3 (*Chrysops* spp.). The *cox*1 sequence of *Acalles guadarramaensis* (GenBank: GU988096) was used as the out-group (Fig. 2). *cox*1 sequences of tabanids in the present study were deposited in the GenBank database under the accession numbers MW013783-MW013793.

### Molecular characterization and phylogeny of hosts

Molecular and phylogenetic analysis of mitochondrial *16S* rRNA genes amplified from the blood meal of tabanids using vertebrate-specific primers identified *Homo sapiens* as the host of *A. fuscipes*, *C. distincipennis* and *T. taeniola*. The hosts of *T. guineensis* were *C. sabeus* and *H. sapiens*. The results obtained showed that 96.43% of all tabanids analysed had their *H. sapiens* meals compared to 3.57% for *C. sabeus*.

### Molecular detection, identification and phylogenetic analysis of pathogens

#### Detection of *Trypanosoma* spp.

**Fig. 3:**
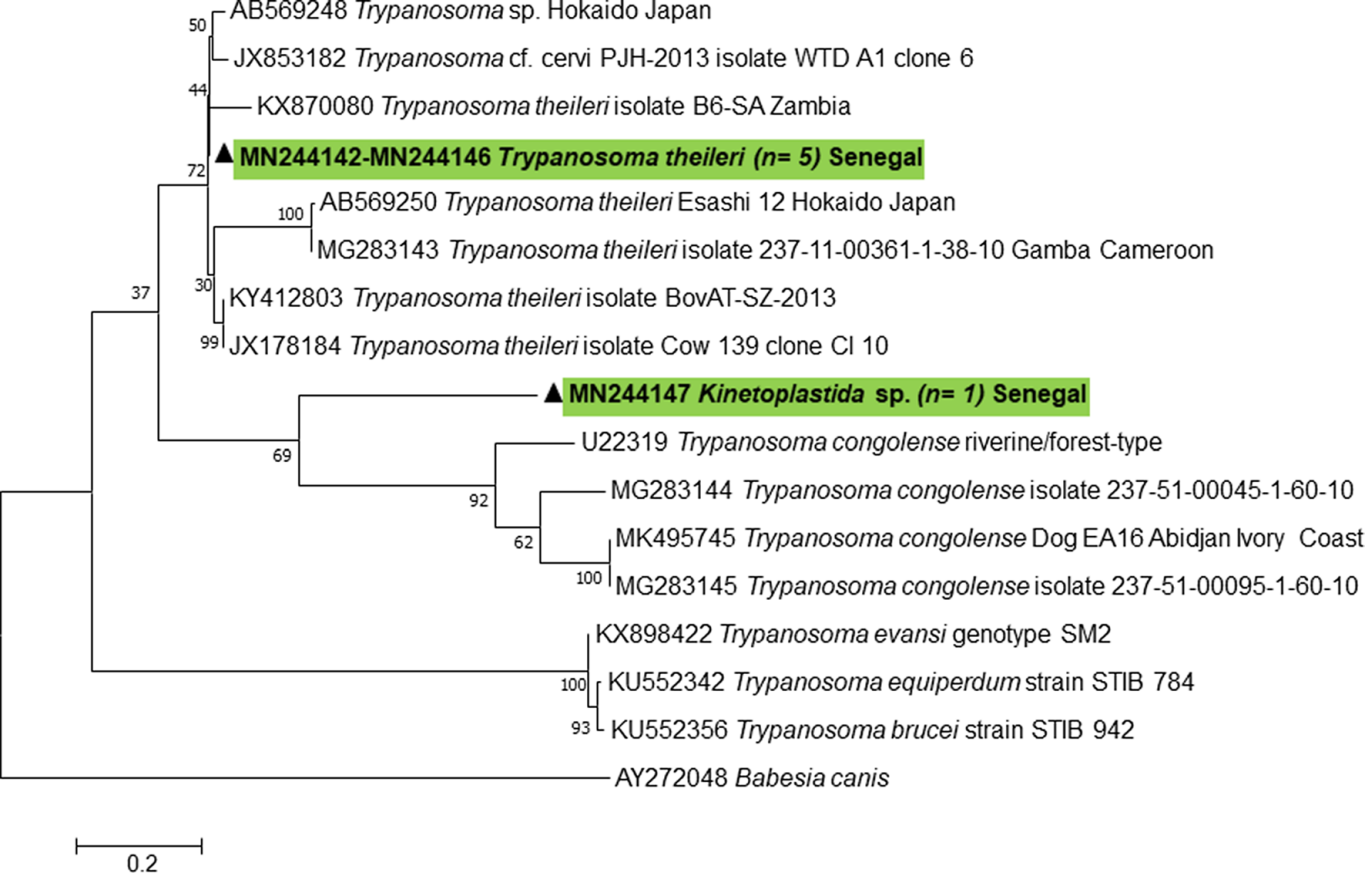
Maximum likelihood phylogeny of *Trypanosoma theileri* and Kinetoplastida sp. detected on tabanids in the present study. The evolutionary history, based on the ITS1 region, was inferred by using the maximum likelihood method based on the Tamura 3-parameter model. The tree is drawn to scale, with branch lengths measured in the number of substitutions per site. The analysis involved 17 nucleotide sequences. All positions containing gaps and missing data were eliminated. There were a total of 206 positions in the final dataset

Among the analysed samples, 9/136 (6.6%) *A. fuscipes* were positive for *Trypanosoma* spp. by qPCR for the *5.8S* gene (Table 2). Amplifications were obtained for the *28S* and *18S* genes (550 bp and 600 bp, respectively) using Kinetoplastida-specific primers; and for the ITS1 region (250–710 bp) using *Trypanosoma* spp.-specific primers. No positive amplification was found in the other tabanid species (Table 2). Sequencing revealed that 8 of these samples presented 97% identity for the *28S* gene with *T. minasense* (GenBank: AB362411), > 99% identity with *T. theileri* (GenBank: KR024688; LC385952) for the *18S* gene and 92–93% identity with *Trypanosoma* sp. (GenBank: AB569248) and *T. theileri* (GenBank: KX569248) for the ITS1, respectively. One sample (TBC 05) exhibited divergent similarity for the ITS1 sequence with the other trypanosomes in the GenBank database, but sequencing for the other genes (*18S* and *28S*) was not possible. The sequences of the specimen were of remarkable specificity. The phylogenetic analysis based on ITS1 sequences (Fig. 3) showed the formation of two clusters, one authentic *T. theileri* cluster and another cluster atypical of all GenBank kinetoplastid sequences. *Homo sapiens* has been identified as the host of all positive samples for *Trypanosoma* spp. Sequences were deposited in the GenBank database under the accession numbers MN244142-MN244147 (ITS1); MN244154-MN244159 (*28S*) and MN244160-MN244163 (*18S*).

**Table 2:** Summary of pathogen research results

Pathogen	*Atylotus fuscipes* (*n* = 136)			*Chrysops distinctipennis* (*n* = 6)			*Tabanus taeniola* (*n* = 1)			*Tabanus guineensis* (*n* = 28)			Total (*n* = 171)
	qPCR *n* (%)	PCR *n* (%)	BLAST *n* (%)	qPCR *n* (%)	PCR *n* (%)	BLAST *n* (%)	qPCR *n* (%)	PCR *n* (%)	BLAST *n* (%)	qPCR *n* (%)	PCR *n* (%)	BLAST *n* (%)	qPCR *n* (%)
*Trypanosoma* spp.	9 (6.6)	9 (6.6)	*T. theileri*	0 (0)	–	–	0 (0)	–	–	0 (0)	–	–	9 (5.26)
*Leishmania* spp.	9 (6.6)	4 (2.9)	*L. donovani*	3 (50.0)	0 (0)	–	1 (100)	0 (0)	–	10 (35.7)	2 (7.1)	*L. donovani*	23 (13.4)
*Filaria*	2 (1.47)	2 (1.5)	*S. digitata*	0 (0)	–	–	–	–	–	1(3.5)	1 (3.57)	*S. digitata*	3 (1.7)
*Rickettsia* spp.	7 (5.1)	4 (2.7)	*R. slovaca*; *R. africae*; *R. montanensis*	0 (0)	–	–	0 (0)	–	–	0 (0)	–	–	7 (4.1)
*Anaplasmataceae*	1 (0.7)	0 (0)	–	1(16.7)	0 (0)	–	0 (0)	–	–	5 (17.8)	0 (0)	–	7 (4.1)
*Coxiella burnetii*	0 (0)	–	–	0 (0)	–	–	0 (0)	–	–	0 (0)	–	–	0 (0)
*Mansonella* spp.	0 (0)	–	–	0 (0)	–	–	0 (0)	–	–	0 (0)	–	–	0 (0)
Piroplasmida sp.	0 (0)	–	–	0 (0)	–	–	0 (0)	–	–	0 (0)	–	–	0 (0)

*Abbreviations*: *n*, number of samples by species; (%), percentage positive; –, not realized

#### Detection of *Leishmania* spp.

**Fig. 4:**
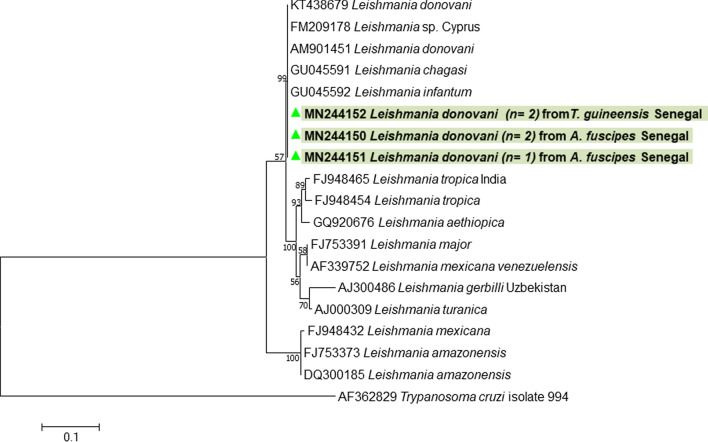
Molecular phylogenetic tree showing the position of the *Leishmania donovani* complex identified in the present study according to sequences from GenBank. The evolutionary history, based on the ITS1 region, was inferred by using the maximum likelihood method based on the Tamura 3-parameter model. The tree is drawn to scale, with branch lengths measured in the number of substitutions per site. The analysis involved 19 nucleotide sequences. All positions containing gaps and missing data were eliminated. There were approximately 300 positions in the final dataset

Molecular detection of *Leishmania* showed that 13.45% (23/171) of tabanids, consisting of 9/136 (6.61%) samples of *A. fuscipes*, 3/6 (50%) of *C. distingutipennis*, 1/1 of *T. taeniola* and 10/28 (35.7%) of *T. guineensis*, were positive by qPCR. Only 4/9 positive samples of *A. fuscipes* and 2/10 positive samples *T. guineensis* were positive by standard PCR for the ITS1 region (480 bp) and the amplicons were successfully sequenced (Table 2). The obtained sequences were deposited to the GenBank database under the accession numbers MN244150-MN244153. BLAST and phylogenetic analysis confirmed the presence of *L. donovani* (GenBank: KU680955) (Fig. 4). *Homo sapiens* was identified as host for the tabanids positive to *Leishmania* spp.

#### Filaroid nematode detection

**Fig. 5:**
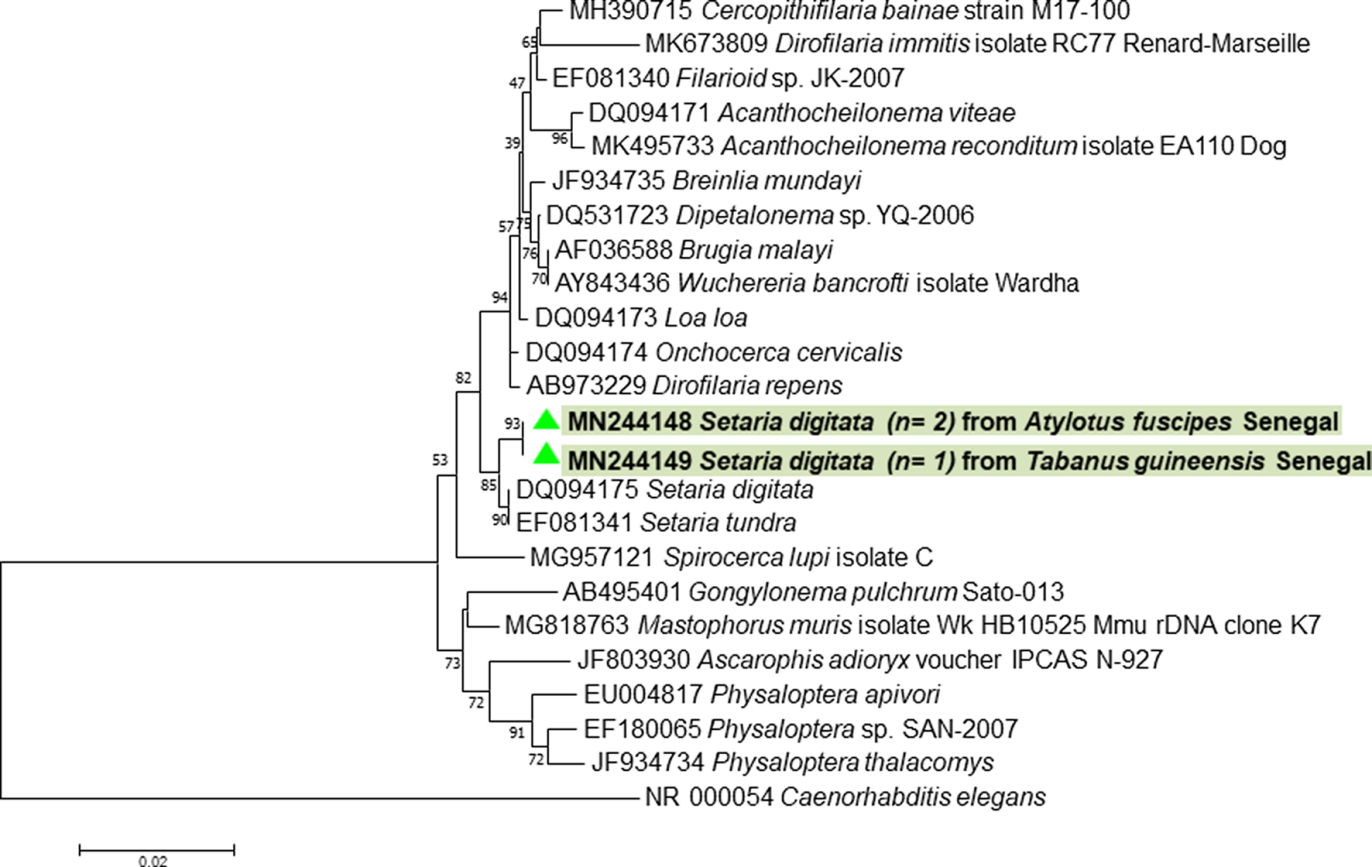
Phylogenetic analysis showing the position of *Setaria digitata* detected in the present study. The evolutionary history based on *18S* rRNA gene was inferred using the neighbour-joining method. The evolutionary distances were computed using the Tamura-Nei method and are in units of the number of base substitutions per site. The differences in the composition bias among sequences were considered in evolutionary comparisons. The analysis involved 24 nucleotide sequences. All positions containing gaps and missing data were eliminated. There were a total of 708 positions in the final dataset

The search for filaria yielded, by qPCR for *28S* and standard PCR for *18S*, 2 positive samples out of 136 (1.5%) *A. fuscipes* and 1/28 (3.5%) *T. guineensis* (Table 2). Phylogenetic analysis confirmed the detection of *S. digitata* (GenBank: DQ094175) (Fig. 5). These sequences were deposited to the GenBank database under the accession numbers MN244148-MN244149. The host identified for all *Setaria digitata*-positive samples was *H. sapiens*.

#### *Rickettsia* spp. detection

**Fig. 6:**
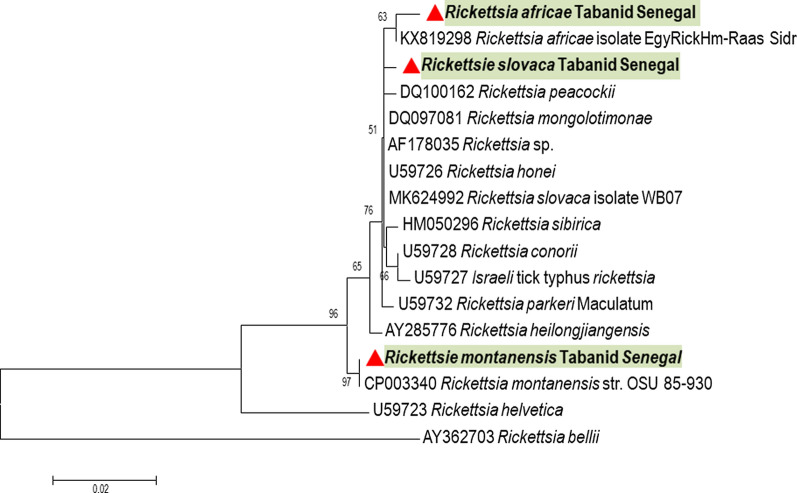
Molecular phylogenetic analysis for *Rickettsia* spp. detected on Senegalese tabanids. The evolutionary history based on the *gltA* gene was inferred by using the maximum likelihood method based on the Tamura 3-parameter model. The tree is drawn to scale, with branch lengths measured in the number of substitutions per site. The analysis involved 17 nucleotide sequences. All positions containing gaps and missing data were eliminated. There were a total of 441 positions in the final dataset

Among all the tabanids tested, only *A. fuscipes* was positive for *Rickettsiae*, with 7/136 (5.1%) samples by qPCR. Four of them were amplified by nested PCR and successfully sequenced (Table 2). Two obtained sequences were nearly identical (99.33%) to *R. slovaca* isolate WB07 (GenBank: MK624992). One sequence had 99% identity with *R. africae* (GenBank: KX819298), which was detected on *Hyalomma marginatum* (camel tick) from Egypt [29]. Another sequence showed 100% similarity with *R. montanensis* str. OSU 85-930 (GenBank: CP003340) (Fig. 6).

#### Detection of Anaplasmataceae

Using a *23S*-based qPCR, we detected DNA of *Anaplasmataceae* in 1/136 (0.73%) *A. fuscipes*, 1/6 (16.66%) *C. distinctipennis* and 5/28 (17.85%) *T. guineensis*. The only specimen of *T. taeniola* tested negative (Table 2). We were unable to identify the positives by *23S*-based standard PCR.

### MALDI-TOF/MS analysis

**Fig. 7:**
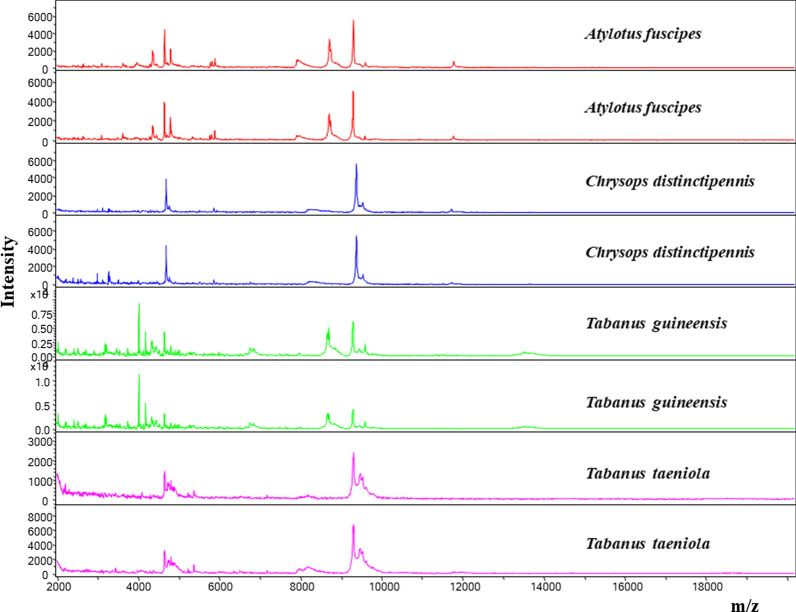
MALDI-TOF specific spectra for four species of Senegalese tabanids

A total of 22 specimens, including 14 *T. guineensis*, 6 *C. distinctipennis*, one *T. taeniola* and one *A. fuscipes*, were submitted for MALDI-TOF MS analysis. We obtained good quality spectra for 20 specimens and poor quality for 1 one *C. distinctipennis* and one *T. taeniola*. Intraspecies reproducibility and species specificity by analysing MS spectra obtained from different species using Flex analysis software are shown in Fig. 7. The blind test showed that the 16 specimens with good quality spectra (12 *T. guineensis* and 4 *C. distinctipennis*) were correctly identified, with scores ranging from 1.800 to 2.519.

## Discussion

In this period, marked by the increasing success of genomics, the molecular and proteomic identification of Senegalese tabanids as vectors of pathogens is a reliable approach. Indeed, this approach makes it possible to further characterize these important arthropods, to better define their roles in the transmission of pathogens and to improve the tools intended for their elimination.

All of the specimens captured in this study were females maybe due to the quality of the trap used in Casamance. In Niokolo-Koba, capture of females only was a coincidence. Ovazza & Valade [25], observing the diurnal activity of certain tabanid species in Senegal, reached the same conclusions. In addition, the haematophagous nature of females with a preference to draw their meals on humans or animals, particularly domestic ones, could also explain these findings [30].

According to the results, there are more samples and greater species diversity in Casamance than in Niokolo-Koba, probably due to the lengths of the capture period (one week in Casamance and one day in Niokolo-Koba) and the capture methods. We should also consider the park of Niokolo-Koba, a wildlife reserve, with low human density. In agreement with Ovazza [30], the uniqueness of the specimen of *T. taeniola* we captured could be explained by the fact that this species is more frequently harvested by day than by night on cattle and humans.

In the present study, four species were morphologically characterized, i.e. *A. fuscipes*, *C. distingutipennis*, *T. taeniola* and *T. guineensis*. Hamon et al. in 1956 [31], Ovazza in 1967 [30] and Raymond et al. in 1980 [22] reported in separate works their existence in Senegal. The description of specimens at the species level performed in our study was consistent with that of Ricardo [4] and Crosskey [28] for *T. guineensis*, Mugasa et al. [7] for *C. distingutipennis* and Baldacchino et al. [2] and Hamon et al. [31] and Al-Talatha et al. [1] for *A. fuscipes*.

The molecular identification revealed three species, i.e. *A. fuscipes*, *C. distinctipennis* and *T. guineensis*. To date, there are no sequences of *A. fuscipes* in the GenBank database. However, phylogenetic analysis involving *cox*1 sequences of several *Atylotus* species has shown that *A. fuscipes* is distinct from *A. diurnus* and *A. nigromaculatus* described in East Africa. It exhibited 99% identity with them. To our knowledge, this is the first study where genomic sequences were obtained for *A. fuscipes*. It seems that morphological differences in the genus *Atylotus* are not supported by genetic differences.

The sequences of *C. distingutipennis* had up to 98% identity with those in GenBank (KX946519) [7]. BLAST analysis of *T. guineensis* sequences against those described by Mugasa et al. [7] in Kenya (GenBank: KX946537, KX946566) showed an identity of only 93%. Phylogenetic analysis revealed two distinct clades, confirming that they most likely represent two genetically different species. Based on arguments from the literature and phylogeny, it appears that our samples are more likely to be authentic *T. guineensis* than species captured in Kenya, as the holotypes and lectotypes described in 1908 by Ricardo [4] and again in 1963 by Crosskey [28] originated in West Africa (Nigeria and Guinea). In addition, according to Oldroyd [24], the distribution of this species is limited exclusively to West Africa.

Proteomic analysis by MALDI-TOF MS made it possible to determine, to the best of our knowledge, for the first time, specific and reproducible spectra of four tabanid species, *A. fuscipes*, *C. distingutipennis*, *T. guineensis* and *T. taeniola*. Differential peaks were found to be distinct by species (Fig. 7). However, not without being unusable, *T. taeniola* spectra were slightly defective. The uniqueness of the specimen collected made it impossible to repeat the tests.

Since this study is the first proteomics analysis of tabanids by MALDI-TOF MS, it will not be possible to compare its results here. However, the data provided in our study may establish a foundation for future work concerning the identification of tabanid flies by MALDI-TOF MS.

The analysis of the blood meals revealed that *H. sapiens* is the principal host of the tabanids from Casamance (*A. fuscipes*, *C. distinctipennis* and *T. taeniola*). The hosts of the tabanids from Niokolo-Koba (*T. guineensis*) were *H. sapiens* (96.42%) and *C. sabeus* (3.57%). These two host types occupied different ecosystems, specifically the village agglomerations of Casamance for the first and Niokolo-Koba Park for the second. In the present study, humans were identified as the main source of blood meals for the tabanid females in Senegal. The host preference cannot be established conclusively, and we suggest that this finding is most likely due to sampling sites. Ovazza [30] also found similar results. The exploration of other sites can reveal more hosts. However, we may not exclude that the identification of humans as associated hosts may be influenced by some possible sample contamination during collection.

In this study, *C. sabeus*, a non-human primate, was characterized as a host of tabanid flies for the first time. Previously, in a study conducted in Congo, Gouteux et al. [28] found non-human hosts, including the hippopotamus, ruminants and lizards. These results suggest possible transmissions by tabanids of pathogens harboured by these hosts.

Pathogens detected in *A. fuscipes* belonged to the Kinetoplastida: *T. theileri*; a high percentage of the *L. donovani* complex; and an unknown Kinetoplastida sp. The obtained *28S* rDNA sequences of *T. theileri* exhibited 97% similarity with *T. minasense* (GenBank: AB362411). This finding is due to the absence of *28S* rDNA sequences for *T. theileri* on GenBank. *Trypanosoma theileri* has been confirmed by amplifying the *18S* gene and the ITS1 region. The existence of the *L. donovani* complex in tabanids has already been mentioned [32], but its involvement in the transmission cycle and the occurrence of disease has not been determined. Our study could not provide an explanation for the strong preponderance of *Leishmania* among Senegalese tabanids. This molecular detection could also be attributed to the fact that tabanids feed on infected animals/humans and cannot maintain pathogen multiplication [33]. However, visceral leishmaniosis (VL) cases reported in West Africa are rare compared to other African endemic regions, such as north and eastern Africa. In Senegal, high seroprevalence of canine leishmaniasis (> 40%) confirmed by PCR and more than 30% seropositive people have been reported [34]. Niger reported 21 cases, Côte dʼIvoire reported eight cases, and Gambia and Burkina Faso reported one case each. Most of these cases occurred more than 10 years ago; one Togolese case was diagnosed in Lama-Kara (northern region) in 1994; and in Nigeria, suspected cases were reported between 1936 and 1947 [35], and 60 cases were reported between 2005 and 2012 [36]. A recent survey in Ivorian dogs showed a seroprevalence of 15%, and *L. infantum* DNA was present in 4% of dogs [37]. On the other hand, VL vectors are not described in the literature. *Phlebotomus orientalis* and *P. alexandri*, the confirmed vectors of *L. donovani* and *L. infantum*, respectively, have not been identified in Senegal, Côte dʼIvoire, Mali, and Burkina Faso [38–40]. Experimental models are required to clarify this ambiguity concerning the *Leishmania*-vectorial role of tabanids.

Phylogenetic analysis based on the ITS1 region of the detected trypanosome sequences showed that the specimen named Kinetoplastida sp. was most likely a new species of *Trypanosoma* sp. (Fig. 3). Studies had previously described *T. theileri* as pathogens in tabanids [9, 41–43]. Similarly, the presence of *T. theileri* in Casamance was noted by Touré [26] in a study describing this species. A high (25%) prevalence of *T. theileri* in the studied horse flies was detected in several West African countries [44]. Investigating trypanosomes in Senegal is of great interest in the prevention and control of human and especially animal trypanosomiasis in view of the size of the livestock available in this country. Other protozoans, such as piroplasmids, were not detected in the present study.

A nematode belonging to the family Setaridae was detected in *T. guineensis* and *A. fuscipes*. It is well-known that filariae, such as *Loa loa*, are transmitted by tabanids [9, 43], but *S. digitata*, mainly recognized as the aetiological agent of animal filariasis, has not been investigated in tabanids. The present study demonstrates the existence of a new genus of the Tabanidae, including *Atylotus* (*A. fuscipes*), as potential carriers of wireworms. To date, only *Chrysops* spp. were known as worm vectors in the Tabanidae. The revelation concerning the presence of filariae in Casamance and Niokolo-Koba provides important data for understanding the many filariases that affect human populations and their animals in Senegal. This research is part of the government’s efforts to prevent and control locusts. In the past, only two species of *Onchocerca* spp. has been reported in the cattle of Kedougou by Vassiliades et al. [21]. Finally, in the present study, positive tabanids for the different pathogens had *H. sapiens* as the principal host.

We detected bacteria belonging to the family *Anaplasmataceae* and to the genus *Rickettsia*. We were not able to amplify and characterize *Anaplasmataceae*-positive samples, probably due to the low bacterial load reflected by the high cycle threshold (34–36) found by qPCR. Several bacterial species have been detected in tabanids [2, 45]. *Tabanus* spp. and *Chrysops* spp. have been reported as mechanical vectors of *Anaplasma marginale*, while *C. vittatus* and *Tabanus* spp. were recognized as vectors for *Ehrlichia risticii* [2]. In this study, DNA of *Anaplasmataceae* was detected in *A. fuscipes*, *C. distinctipennis* and *T. guineensis.* In addition, three *Rickettsia* genotypes have been detected in *A. fuscipes.* Unfortunately, we could not better characterize the identified rickettsial species because of the low bacterial load in the samples studied. Senegal, however, is an endemic area for *Anaplasmatacea* [45] and *Rickettsia* spp. [46] infections. In the same study area, Mediannikov et al. [47] detected a new *Rickettsia* sp. in tsetse flies. *Anaplasma marginale* and rickettsial endosymbionts have been molecularly identified in tabanids from Hungary [48]. The role of tabanids as vectors of *Anaplasmatacea* and/or *Rickettsia* spp. has not been characterized to date. In addition, no *Coxiella burnetii* DNA was detected, while *C. burnetii* has already been detected in *T. staegeri* and was not captured in the present study [2].

## Conclusions

Previously overlooked and therefore insufficiently examined, tabanids from Senegal in particular and from West Africa in general, are nevertheless potential carriers of multiple pathogens. Investigating these dipterans using innovative tools and genomic and proteomics methods is of considerable interest to animal and human public health. Several limitations to this research are notable, particularly with regard to the duration and uniqueness of some specimens characterized in this study. However, this study represents a strong contribution to the understanding and prevention of vector-borne diseases caused by tabanids in West Africa. Furthermore, this report is the first to describe the diversity among Senegalese tabanids, their hosts and associated pathogens. The molecular results and sequences of *A. fuscipes* and *T. guineensis* and the presence of *L. donovani*, *Trypanosoma* spp., *S. digitata* and *Rickettsia* spp. are notable. To thoroughly characterize these species, it is important that studies in entomology and infectiology be pursued to achieve the molecular characterization and MALDI-TOF MS analysis of tabanids from Senegal and West Africa. These efforts, if realized, may enrich the genomic and proteomic databases for tabanids. Screening for other pathogens not studied in this research is equally important. Finally, experimental models should be considered to better understand the ability and competence of Senegalese tabanids to carry or transmit the pathogens detected in this study.

## Data Availability

All data are included in the manuscript. The newly generated sequences were deposited in the GenBank database under the accession numbers: MW013783-MW013793 (*cox*1) of tabanids; MN244142-MN244147 (ITS1); MN244154-MN244159 (*28S*) and MN244160-MN244163 (*18S*) of *Trypanosma* spp., MN244150-MN244153 (ITS1) of *Leishmania* spp., MN244148-MN244149 (*18S*) of *Setaria digitata*.

## Abbreviations

MALDI-TOF MS: Matrix assisted laser desorption ionization-time of flight mass spectrometry
PCR: Polymerase chain reaction
